# The λ Red Proteins Promote Efficient Recombination between Diverged Sequences: Implications for Bacteriophage Genome Mosaicism

**DOI:** 10.1371/journal.pgen.1000065

**Published:** 2008-05-02

**Authors:** Jann T. Martinsohn, Miroslav Radman, Marie-Agnès Petit

**Affiliations:** 1 Faculté de Médecine R. Descartes, INSERM U571, Université Paris Descartes, Paris, France; 2 INRA, UR888, Jouy en Josas, France; Baylor College of Medicine, United States of America

## Abstract

Genome mosaicism in temperate bacterial viruses (bacteriophages) is so great that it obscures their phylogeny at the genome level. However, the precise molecular processes underlying this mosaicism are unknown. Illegitimate recombination has been proposed, but homeologous recombination could also be at play. To test this, we have measured the efficiency of homeologous recombination between diverged *oxa* gene pairs inserted into λ. High yields of recombinants between 22% diverged genes have been obtained when the virus Red Gam pathway was active, and 100 fold less when the host *Escherichia coli* RecABCD pathway was active. The recombination editing proteins, MutS and UvrD, showed only marginal effects on λ recombination. Thus, escape from host editing contributes to the high proficiency of virus recombination. Moreover, our bioinformatics study suggests that homeologous recombination between similar lambdoid viruses has created part of their mosaicism. We therefore propose that the remarkable propensity of the λ-encoded Red and Gam proteins to recombine diverged DNA is effectively contributing to mosaicism, and more generally, that a correlation may exist between virus genome mosaicism and the presence of Red/Gam-like systems.

## Author Summary

Temperate bacterial viruses alternate between a dormant state, during which viral DNA remains integrated in the host genome, and a lytic state of phage multiplication. Temperate viruses have a characteristic genome organisation known as ‘mosaic’ – they contain ‘foreign’ segments that originate from related viruses. In pairwise alignments between a given virus and its relatives, the overall nucleotide sequence identity is around 50%. In contrast, the mosaic segments are 90% to 100% identical. How mosaics are generated is largely unknown, but it is likely that related viruses meet in the same bacterium and undergo random recombination, with emergence of the most robust recombinatory viruses. The prevalent hypothesis is that mosaics are formed by illegitimate recombination. We propose and demonstrate that an alternative driving mechanism, homologous recombination, is used for mosaic formation between similar but diverged viral sequences. Using the well known *Escherichia coli* λ virus as a paradigm, we show that such homeologous recombination is remarkably efficient. This finding has important implications in the field of virus genome evolution, as it may explain the high plasticity of viral genomes. It is also applicable to the field of biotechnology, and reveals viruses to be promising vectors for shuffling genes *in vivo*.

## Introduction

Bacterial viruses (bacteriophages) are the most abundant and diverse life form and exhibit high levels of evolvability and adaptability [1]. Moreover, bio-informatic studies suggest that they contribute substantially to bacterial genome evolution. For example, in γ Proteobacteria, most genes unique to a particular bacterial species or to a taxonomic group of species, are relatively short and AT-rich–two hallmarks of phage genes [2].

A particularity of temperate virus genome evolution is their extensive sequence mosaicism [3] due to exchange of DNA sequences, facilitated by the frequent encounter inside the same bacterial host, for example between an invasive and a resident virus [4],[5]. However, most of the time, this mosaicism does not perturb the general gene order (synteny), probably due to counterselection of suboptimal gene combinations [6].

Little is known about the precise molecular processes underlying this viral genome mosaicism. In the case of fully sequenced lambdoid viruses isolated from enterobacteria, genomes are on average 50% identical, except for DNA sequence patches showing more than 90% identity. The apparent absence of any particular signals at the borders of sequence-similar patches has led to the proposal that they have probably been acquired by illegitimate recombination [7],[8]. In some cases however, exchange of sequence modules can be explained by homologous recombination involving flanking, short and conserved sequences shared by a subset of related viruses [9]. But it is also possible that some regions flanking the most similar shared sequences have undergone homeologous recombination, *i.e.* recombination between related but diverged DNA sequences [10].

The temperate virus λ has been a major model system in classical molecular genetics, including in the study of homologous recombination, which occurs at high rates in the λ genome (reviewed by [11]). λ encodes its own homologous recombination genes *red*α, *red*β (the Red system) and the *gam* gene, all belonging to the pL-operon. Redα is a double strand specific 5′ to 3′ exonuclease [12] and Redβ mediates strand annealing and exchange reactions starting from DNA extremeties [13]. The λGam protein inactivates the *E. coli* exonucleaseV (RecBCD), thereby protecting the ends of its linear genome from degradation (reviewed by [14]). Furthermore two other genes in the *nin* region participate in Red-mediated recombination: the *orf* gene product can replace the three proteins RecFOR involved in the *E. coli* RecF recombination pathway, and the *rap* gene codes for a Holliday junction resolvase [15]–[18]. Intracellular λ DNA is substrate for both virus-encoded and *E. coli* host recombination machineries, i.e., λ^+^ recombines well in a *recA* host and so does λ *red gam* in the Rec^+^ host if it contains a Chi site to resist RecBCD degradation. In both cases, most events are non-reciprocal [19],[20]. For both RecA-dependent and Red-dependent recombination, the required minimal homology is around 30 bp [21],[22].

To test the efficiency of recombination between diverged sequences in viruses, we have investigated the capacity of λ to recombine pairs of homeologous *oxa* genes, starting from a λ strain initially described by the group of Kleckner [23] and later examined in greater detail by Ennis *et al*. [24]. In this system, recombination between inverted repeats framing the pL promoter leads to its inversion, which is accompanied by a phenotypic switch. We observed that the *Escherichia coli* RecABCD pathway recombined 22% diverged genes with a frequency of 10^−6^ per virus generation. Interestingly, the λ Red pathway showed a 100-fold higher efficiency. The recombination editing proteins MutS [25], UvrD [26] and RecQ [27] had only marginal, if any, effect on λ recombination. Sequences of genes resulting from homeologous recombination revealed a broad spectrum of hybrids, and some differences between the products generated by the Rec and the Red proteins which may reflect intrinsic properties of the two recombination pathways. Therefore this λ system provides an efficient “gene machine” to create large libraries of hybrid sequences for biotechnology applications. In an attempt to assess the contribution of homeologous recombination between diverged sequences to phage mosaicism, we undertook a systematic bioinformatic analysis of mosaic flanking sequences, in three families of lambdoid phages. We found that half of them had at least one moderately diverged flanking region. This suggests that homeologous recombination within such flanking sequences may facilitate the reshuffling of phage genome modules and underlines the important role of virus recombination proteins in their genome evolution.

## Results

### λ Promotes Efficient Recombination between Diverged Sequences

**Figure 1:**
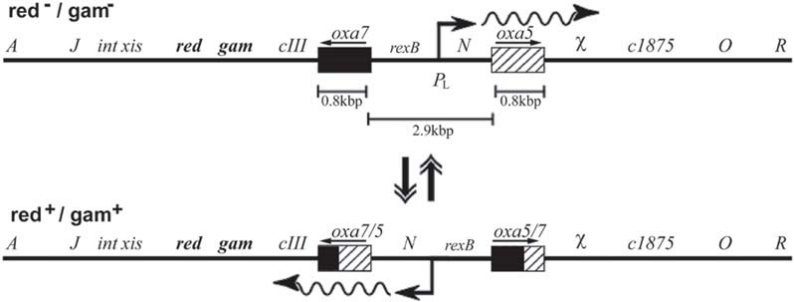
Recombination between the two homeologous *oxa* sequences flanking the λ pL promoter leads to a phenotypic switch. In λ *red*- *gam*-, transcription from the pL promoter proceeds rightward and the *red* and *gam* genes are not transcribed. Recombination between the two homeologous sequences leads to inversion of the pL-N segment causing a selectable phenotype (see Text for details). Not drawn to scale.

To test the efficiency of homeologous recombination in virus genomes, we studied recombination between pairs of sequence diverged genes inserted into the genome of λ. The experimental system is based on a genetic switch in λ resulting from homologous recombination between two identical inversely oriented IS10 sequences flanking the promoter pL [23]. Recombination between such inverted repeats is accompanied by the inversion of the pL promoter, leading to a phenotypic switch used to score recombinants (Figure 1). In the normal pL orientation the *red* and *gam* genes are transcribed such that λ grows on a *recA* mutant host, but not on a P2 lysogen. In the opposite orientation of pL, *red* and *gam* are not expressed, and λ grows on a P2 lysogen, but not on a *recA* strain.

Starting from the construct with the inverted pL orientation, the two IS10 sequences were replaced with approximately 800 bp *oxa* genes having different levels of divergence [28]. A properly oriented Chi site was introduced rightward from the recombination cassette, to allow for stimulation of RecBCD-promoted recombination (see Figure 1, alignments of *oxa* genes are shown in Figure S1). Recombinant frequencies at 0%, 4%, and 22% divergence were measured during single step growth on C600(P2). In this background, homologous recombination occurs via the host RecABCD pathway only, because the λ-encoded pathway mediated by Red is repressed, due to the inverted pL promoter, and the Chi site protects λ rolling-circle forms from pure RecBCD degradation. Similarly, using λ with the native pL orientation, frequencies were measured during single step growth on a *recA* host. This time, recombination occurs via the phage-mediated Red pathway only, as the *recA* gene is mutated, and RecBCD is inactivated by Gam. We verified that in both backgrounds, λ replicated by a rolling circle mode (see Methods).

**Figure 2:**
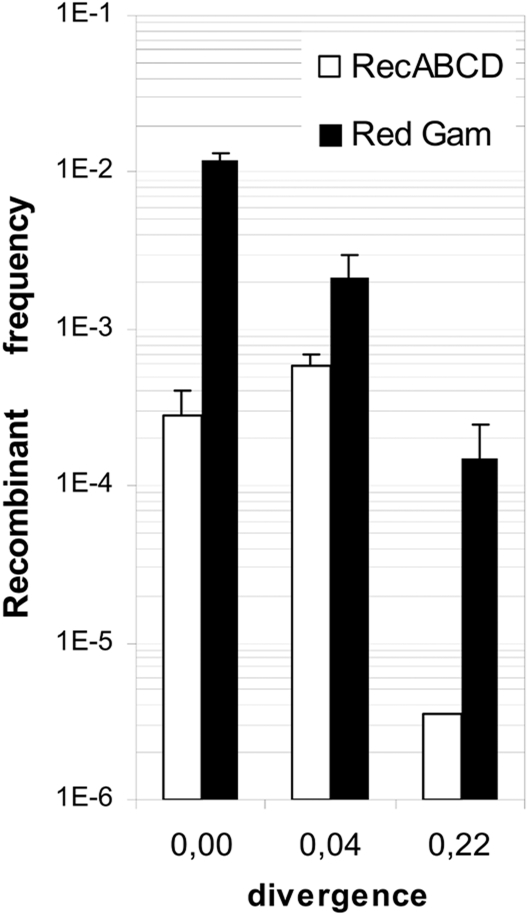
Frequency of recombinants in the RecABCD and the Red pathways, at different levels of sequence divergence, determined by single step growth of phages. Every bar shows the mean and standard deviation deduced from at least three experiments.

In the RecABCD pathway, maximum inversion frequency was 3×10^−4^ for identical sequences, whereas the minimum measured was 3×10^−6^ for 22% divergence (Figure 2). Unexpectedly, the recombinant frequency was as high between 4% diverged sequences as between identical sequences, and this effect persisted in the *mutS* background (see Table 1). A 4% divergence was reported to reduce by 1000 fold homologous recombination in the *E. coli* chromosome [29]. No obvious sequence stimulating recombination, such as a Chi site, is present in the *oxa11* sequence used to construct the 4% diverged substrate. Rather than being a stimulation of recombination between 4% diverged sequences, it could be that some process inhibits recombination between the strictly identical sequences in λ.

**Table 1:** Recombinant frequency between 0%, 4% and 22% diverged sequences present in λ, in the RecABCD and the Red pathway.

	0% divergence^a^					
	RecABCD pathway		fold/WT	Red pathway		fold/WT
WT	3,5×10^−4^	±2,8×10^−4^		7,3×10^−3^	±2,2×10^−3^	
*mutS*	3,3×10^−4^	±2,7×10^−5^	1,0	5,5×10^−3^	±4,3×10^−3^	0,8
*uvrD*	9,8×10^−5^	±1,2×10^−5^	0,3	4,0×10^−3^	±1,2×10^−3^	0,6
*recQ*	1,4×10^−4^	±5,5×10^−5^	0,4	5,3×10^−3^	±7,9×10^−4^	0,7

	4% divergence					
	RecABCD pathway		fold/WT	Red pathway		fold/WT
WT	4,2×10^−4^	±1,8×10^−4b^		1,1×10^−3^	±1,5×10^−4^	
*mutS*	3,4×10^−3^	±2,7×10^−4b^	8,1*	3,9×10^−4^	±3,5×10^−4^	0,4
*uvrD*	2,0×10^−3^	±6,3×10^−4^	3,7	8,0×10^−4^	±2,6×10^−4^	0,7
*recQ*	6,6×10^−4^	±3,8×10^−5^	1,2	7,9×10^−4^	±3,3×10^−5^	0,7
*ruv*	5,0×10^−4^	±6,7×10^−5^	0,9	NT		

	22% divergence					
	RecABCD pathway		fold/WT	Red pathway		fold/WT
WT	8,7×10^−7^	±1,3×10^−7^		9,0×10^−5^	±2,4×10^−5^	
*mutS*	1,9×10^−6^	±1,0×10^−7^	2,2*	1,0×10^−4^	±5,0×10^−5^	1,1
*uvrD*	6,8×10^−7^	±4,6×10^−7^	0,8	1,9×10^−4^	±4,3×10^−5^	2,1
*recQ*	2,3×10^−7^	±3,9×10^−8^	0,3*	8,3×10^−5^	±2,1×10^−5^	0,9
*ruv*	2,5×10^−8^	±1,1×10^−8^	0,03*	3,9×10^−5^	±1,4×10^−5^	0,4

a Unless otherwise stated, average and standard deviation for at least three experiments is shown.

b Average and standard deviation of six experiments.

Difference relative to the wild type (WT) significant at 1% level with a Student test.

Recombination by the phage Red pathway was more efficient than recombination by the RecABCD pathway, especially for 22% divergence (1,5×10^−4^ versus 3,5×10^−6^, Figure 2). However, no recombinant was obtained by the Red pathway within 52% diverged sequences (less than 10^−8^).

### Host Recombination Editing Functions Have Little Impact on Virus Homeologous Recombination

In order to measure recombinant frequencies in various genetic backgrounds, a protocol involving growth of bacteria on agar plates rather than single cycle liquid growth was chosen. Bacteria were infected with phages at a multiplicity of infection of 0.1 and grown to confluence, in the non-permissive host for growth of recombinants. This counteracted selection effects and revealed recombinants produced at the last generation. Recombinant frequencies in the wild type hosts were found to be consistent with the single step experiments (compare Table 1 with Figure 2).

The methyl-directed mismatch repair (MMR) MutL and MutS proteins, and to a lesser extent MutH and UvrD, inhibit homeologous recombination by preventing DNA exchange between diverged repeated chromosomal sequences [30]–[32] and among entire genomes of related species [25],[33]. In our system, mismatch repair deficiency (*mutS*) had an eight-fold stimulating effect on RecABCD promoted recombination for 4% diverged sequences (Table 1, 4% divergence set, lane ‘*mutS*’, RecABCD pathway). This effect was less pronounced (two-fold) for 22% divergence. No stimulating effect of the *mutS* mutation was detected for recombination catalyzed by the phage Red system (Table 1, Red pathway, ‘*mutS*’ lanes). Thus in this virus assay, mismatch repair operates a modest control on the fidelity of the bacterial, RecABCD pathway, and not at all on the phage Red pathway.

In addition to its role in MMR, UvrD helicase has a distinct activity in preventing homologous recombination, such that in a *uvrD* mutant, recombination between identical sequences is increased, generally by a factor of 10 [25], [34]–[36]. UvrD appears to act directly as an “antirecombinase” by dismantling RecA nucleoprotein filaments [26]. In yeast, homeologous recombination is increased in a *sgs1* mutant, a member of the RecQ helicase family [37],[38], and in *E. coli*, RecQ prevents illegitimate recombination [27]. We therefore tested *E. coli uvrD* and *recQ* mutants for a hyper-recombination phenotype both in RecA-mediated and Red-mediated events. The *uvrD* mutation had no effect on the recombination between identical sequences. However, similarly to *mutS*, it conferred a four-fold increase in recombinant frequency only at 4% divergence and only in RecA-dependent recombination. This suggests that it does not exert its distinct anti-recombinase activity on the λ substrates (Table 1, lanes ‘*uvrD*’). RecQ did not prevent recombination in any of our substrates. Rather, recombination appeared slightly decreased in the *recQ* mutant, on 22% diverged sequences (Table 1, lanes ‘*recQ*’).

### Sequence Analysis of Recombination Products Reveals Different Recombination Mechanisms Operating in the Different Substrates

In our λ constructs, the set of chosen diverged sequences were pairs of *oxa* genes, encoding different beta-lactamases. Depending on recombination end-points, different gene combinations should form. A total of 152 phages scored as recombinants were used for sequencing the hybrid *oxa* copies. In all 304 *oxa* genes sequenced, a hybrid was found. This indicates that recombination indeed took place within the 800 bp of partial homology. Among these, a total of 136 new gene combinations were obtained.

**Figure 3:**
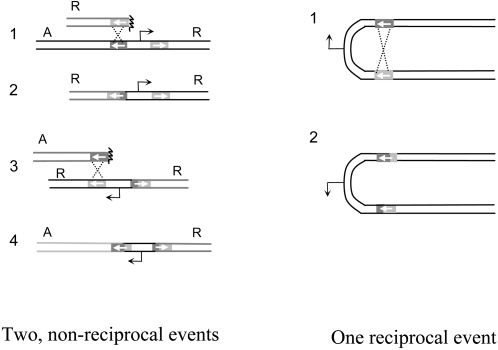
Generation of an inversion between the *oxa* sequences. Left panel: two successive, non reciprocal events: *Oxa* sequences are shown with oriented boxes, dark and light gray. Step 1, a broken piece containing the rightward copy of *oxa* recombines with intact DNA, and generates an unviable molecule (step2), which in turn recombines its leftward *oxa* copy with another *oxa* sequence (step 3). This other broken piece eventually, but not necessarily, consists of the rest of the broken piece shown in step 1. Recombination gives rise to a viable product where the intermediate sequences have been inverted, and the two *oxa* genes are hybrids (step 4). A and R designate the leftmost and righmost genes of λ. Right panel: one reciprocal event. A crossing over is initiated intra-molecularly between the *oxa* sequences (step 1), and gives rise to the inverted configuration (step 2).

The presence of 32 and 176 sites of polymorphism for the *oxa7*-*oxa11* and *oxa7-oxa5* pairs, respectively, allowed us to map precisely strand exchanges and to class recombination events into two main categories: the “non-symmetrical” ones, for which the two joints are present in different intervals, and the “symmetrical” ones, for which the two joints occur in the same interval. Category “complex” includes more complex sequence patterns. Bacteriophage λ recombines essentially in a non-reciprocal mode, but in our recombination assay, only the events that terminate as reciprocal at the DNA level can yield viable recombinants. However, such ‘final’ reciprocity can be reached by two successive non-reciprocal events [39],[40], as shown on Figure 3, left panel. The two events being independent, most products are expected to be of the non-symmetrical category. If, under some conditions, λ recombines in a reciprocal mode at the molecular level, by a simple crossing-over, as shown in Figure 3, right panel, approximately half of the products, those derived from the RuvC-cut strand, are expected to be of the symmetrical category (see Discussion).

For the RecABCD promoted recombination between 4% diverged sequences, most events were non-symmetrical (81%), whereas only 17% were symmetrical (Table 2). Similarly, for the Red promoted events between 22% diverged sequences, a majority (81%) of all events were non-symmetrical and only 17% were symmetrical. In contrast, for the RecABCD promoted recombination between 22% diverged sequences, 55% were symmetrical events, whereas 40% were non-symmetrical events. The difference in the proportions of non-symmetrical events promoted by RecABCD between 4% and 22% diverged sequences was statistically significant as determined by a *Chi* ^2^ test (p<0.0001). Precise positions of the joints in each pair of *oxa* sequence for the 22% diverged DNA are given in Table S1.

**Table 2:** Typology of the pairs of recombination products recovered for 4% and 22% diverged sequences from RecABCD- and Red-dependent events.

	RecABCD pathway				Red pathway	
	4% divergence		22% divergence		22% divergence	
	%	(number)	%	(number)	%	(number)
non-symmetrical	81	(44)	40	(15)	81	(34)
symmetrical	17	(9)	55	(21)	17	(7)
complex	2	(1)	5	(2)	2	(1)
total	100	(54)	100	(38)	100	(42)

Complex recombination products, involving (formally) more than two non-reciprocal events, were observed at similar but low frequencies under all conditions tested.

To test whether the symmetrical events were processed by the RuvABC enzymes, that resolve Holliday junction in a symmetrical way, recombination frequencies were measured in a *ruvABC* mutant strain (Table 1, lanes ‘*ruv*’). Efficiency of recombination between 22% diverged DNA via the RecABCD pathway, was decreased by a factor of 50 in the *ruv* mutant. In contrast, this mutation had no effect on the Red-mediated events for 22% diverged DNA, nor did it affect 4% diverged, RecABCD mediated recombination. Therefore, most of the recombination events observed between 22% diverged DNA in the RecABCD pathway are resolved by Ruv.

**Figure 4:**
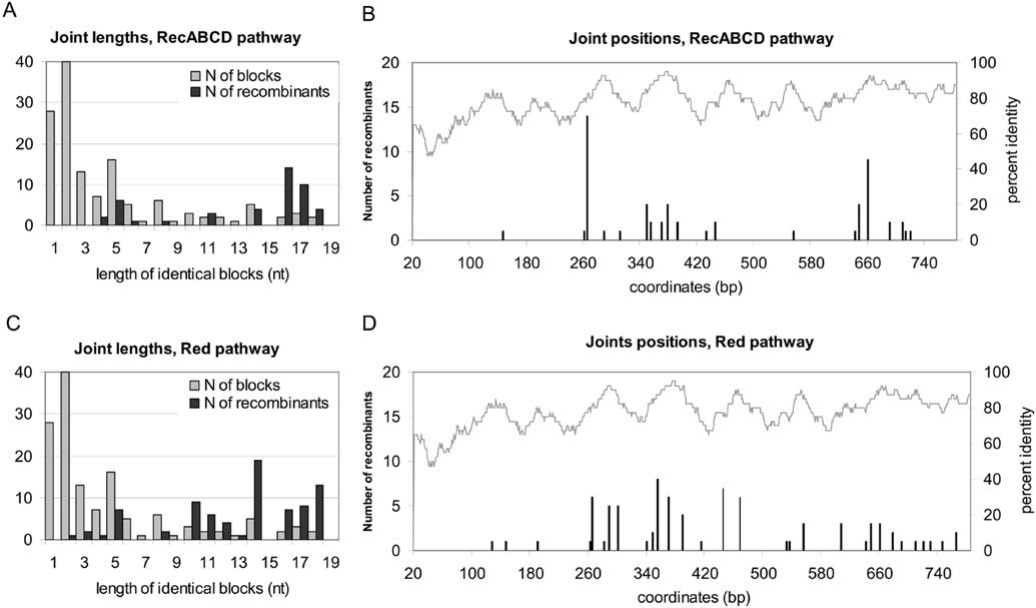
Sequence analysis of recombinant products between 22% diverged oxa. A and C: size distribution of the strictly conserved segments (blocks) of the oxa7/oxa5 alignment (see figure S1, panel B, for the alignment), and number of joints in each size category for recombinants formed in the RecABCD (A) or the Red (B) pathway. B and D: Positional organisation of the joints formed in the RecABCD (B) or the Red (D) pathway along the oxa ORF alignement, 1 is the start position. The upper curve (and the rightward y axis) describes the local percentage of identity along the two sequences, using a sliding window of 40 bp.

Inspection of the location of all recombination joints relative to the length of shared identical sequence blocks revealed, for the 22% diverged sequences, that the joints can occur in regions of homology as small as two bp, but in most cases they were located in the longer identical blocks (Figure 4, A and C). Positions of joints along the *oxa* gene were inspected (Figure 4, B and D), and revealed two preferential blocks for the RecABCD pathway. The first hot spot (nt 266–281, 28% of joints) is 16 nt long and contains two RuvC cutting sites (one on each strand). It may correspond to a preferred resolution site. The second (nt 661–677, 18% of joints) is 17 nt long, does not contain RuvC cutting site, but it is separated by only one mismatch from a 12 bp interval, so that the sum of the two segments is 30 nt, with a 60% GC content, which may help stabilizing the recombination intermediate.

In contrast, the Red pathway did not exhibit such marked hot spots (Figure 4D; the maximal occurrence of a joint was 10%). In both pathways, an overall deficit of joints in the first 260 bp of the gene was observed. It is most likely due to its higher divergence (30% in this segment, versus 18% for the remaining part of the gene, the curve reporting local % identity is drawn above the joints locations in Figure 4 B and D).

In summary, the characteristics of homologous recombination promoted by λ suggest that it may constitute an ideal vector for *in vivo* gene shuffling.

### Detecting Regions of Putative Homeologous Recombination Events in the Natural History of Bacteriophage Genomes

**Figure 5:**
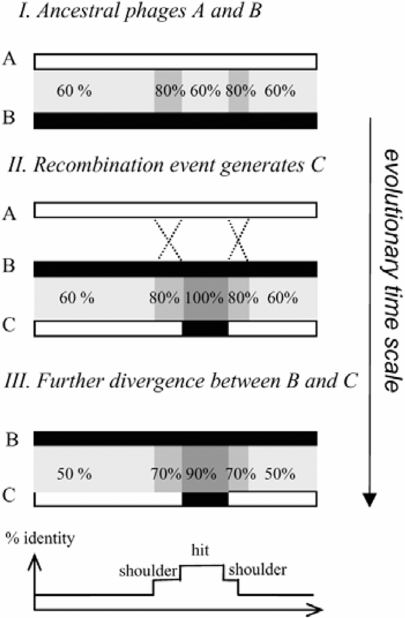
Evolutionary history of event between diverged viruses A and B, produces virus C which is similar to A but for a patch of DNA (hit) coming from B. Alignment of phage B to C reveals at the border of the patch two regions of intermediate similarity (shoulders, here drawn in grey). Below is shown the % identity profile of the B to C virus alignment.

To explore the potential role of homeologous recombination in the evolution of virus genomes, we looked for hallmarks of such events by a comparative bioinformatics analysis of a variety of lambdoid phage genomes. Consider ancestral viruses A and B sharing overall 60% identity except for two 80% identical segments (I, in Figure 5). Homeologous recombination within the 80% identity segments would give rise to phage C consisting of the A sequence with a patch of B. If so, one would expect to find, in the virus C to B alignment, two regions of 80% identity, called hereafter “shoulders”, flanking a patch of 100% identity, called “hit” (II, in Figure 5). Subsequent divergence between ancestral phages B and C would finally lead to 90% identical hits, flanked by 70% shoulders, over a background of 50% identical sequences (III, in Figure 5).

An analysis of ten lambdoid bacteriophages from enterobacteria was performed. It showed that of 83 hits sharing more than 90% identity between any two members of the family, six had two flanking shoulders and 35 a single shoulder. For the remaining 42 hits there was no detectable shoulder (Table 3, first series of data, see Table S2 for the complete data set). To determine the significance of the observed number of shoulders, an estimate of their number expected at random was made. Only seven should have been detected under the random hypothesis, which is six-fold lower than observed and highly significant (p<0.001). The average identity of shoulders was 64% (+/−6.9%) and their lengths were unevenly distributed, with the median of 200 bp (Table 3).

**Table 3:** Analysis of homeologous regions (shoulders) at the border of recombination tracks (hits) in three lambdoid families.

lambdoids growing on		number	% identity		median size (bp)	n exp. shoulders
enterobacteria	hits	83	95	±2,2	400	
(10 genomes)	shoulders	47	64	±6,9	200	7
	hits having 0/1/2 shoulders	42/35/6				
Lactic acid bacteria	hits	193	94	±2,6	500	
(15 genomes)	shoulders	89	66	±6,3	200	27
	hits having 0/1/2 shoulders	113/71/9				
*Staphylococcus aureus*	hits	1321	94	±2,4	500	
(20 genomes)	shoulders	661	68	±6,7	200	249
	hits having 0/1/2 shoulders	745/491/85				

To extend the analysis, hits and shoulders were looked for in 15 lambdoid phages from lactic acid bacteria and 20 lambdoids from *Staphylococcus aureus* (Table 3, last two series, see Tables S3 and S4 for complete data sets). Shoulders were found again in approximately 50% of all hits tested, with a frequency significantly greater than expected at random (p<0.0001 in both cases).

## Discussion

### Red Efficiently Recombines the 22%-Diverged Sequences

The remarkable efficiency of the Red promoted recombination between 22% diverged sequences (10^−4^), in contrast with RecABCD promoted events (10^−6^), can be interpreted in two ways: (i) Redα and Redβ may be less sensitive to sequence divergence during heteroduplex DNA formation than RecABCD, and (ii) Redα and Redβ may escape host factors that prevent RecA-mediated recombination. In support of the first option, Redβ promotes efficient annealing and integration of single strand oligonucleotides containing mismatches, a technique known as recombineering [41]. Redβ is a single strand annealing protein, and has no ATPase activity [42]. It appears therefore as a simpler form of pairing protein as compared to RecA, which may explain its greater tolerance for sequence divergence. It could be also that the two-strand annealing process in Red-promoted events generates mismatched intermediates more readily than the three-strand RecA-promoted D-loops, due to the competition in the latter case with the displaced, and perfectly matched, strand. Interestingly, the related RecE-RecT recombination proteins of prophage *rac* (in a *recBC sbcA* host background) were used successfully to recombine 30% diverged *recA* sequences [43]. Furthermore, recombination between 32% diverged DNA during virus crosses was reported [44]. Finally, RecET promotes recombination between very short sequences (5–13 bp), in a process that may not be very different from the homeologous recombination reported here, albeit less efficient (10^−8^)[45]. Interestingly, in yeast, microhomology-mediated end-joining (MMEJ) depends on Rad52 [46], a protein that has definitely some structural and functional similarities with Redβ and RecT [47].

### λ Escapes from Host Recombination Editing Functions

In support of the other alternative, i.e. the escape from the host recombination editing systems, we have observed that the MutS protein, which prevents RecA-mediated homeologous recombination, is ineffective in the Red pathway. However, MutS can act on Red-mediated single strand annealing [41] excluding the possibility that MutS simply does not detect mismatches generated by Redβ. Actually, even the inhibition by MutS of RecA-mediated homeologous recombination in λ was low (eight fold effect for 4% diverged sequences). In a different but comparable assay, where 4% diverged sequences are recombining in the *E. coli* chromosome, a much more profound, 60 fold inhibiting effect of MutS was reported [29]. It may be that some of the unknown gene products encoded within the λ genome ensure “immunity” against mismatch repair proteins, for instance by inhibiting MutS or MutL. Alternatively the high copy number of λ during the lytic cycle might titrate MutS and/or MutL.

Neither of the helicases UvrD and RecQ showed inhibitory effects on homologous or homeologous recombination, in either the RecABCD or the λ Red pathway (Table 1). Whereas bacterial editing systems act to prevent promiscuous recombination events that cause genome instability, λ virus, and perhaps other lambdoids, appear to evade such editing thereby accelerating the rate of their genome evolution.

### Non-Reciprocal versus Reciprocal Recombination Events

Decades of work and careful analysis of the recombination products in λ crosses have led to the conclusion that in most cases, recombination is non-reciprocal at the molecular level, whether it occurs by the RecABCD pathway, or by the Red pathway [19],[20],[48]. This means in molecular terms that most often, recombination intermediates are not double Holliday junctions resolved by a break-join, RuvABC-dependent process, giving the classical crossover product (as depicted Figure 3, right panel), but are rather one of the three following cases: i) half crossovers resolved by break-join, using either RuvABC or the λ encoded Rap protein [49], ii) D-loops dealt by a break-copy, replication-dependent process, also called BIR [49],[50], or iii) single-strand annealing (SSA) intermediates. The two first situations are compatible with the sketch depicted Figure 3, left panel. The last situation is mostly described for the Red pathway [51],[52].

In the case of our present study, where the recombining sequences are present in λ in inverted orientation in the same molecule, two non-reciprocal events are needed to produce a viable inverted product (Figure 3, left). When the two recombining sequence are diverged, the position of the junction can give a hint of the underlying recombination process. Sequence analysis of pairs of recombinant genes revealed that, in most cases, the junction between the two partner sequences is not at the same position. Thus the hybrid sequence of the two recombined genes is called non-symmetrical, something expected for λ which recombines essentially non-reciprocally. However, half of all RecABCD-promoted recombination between 22% diverged genes showed symmetrical products, i.e. the junctions occurred in the same interval in both copies (Table 2). Because of the abundance of nucleotide polymorphism that define 137 possible intervals for strand exchange, it appears unlikely that two successive events occurred by chance in the same place (probability of 1/137 = 0.7%), and suggests rather that in these cases, recombination occurred by a single crossing-over event. Holliday junction resolution is not expected to give more than 50% symmetrical products in our assay, because the progeny of the two strands of the recombination product is slightly different, due to the difference between the invasion step (not necessarily strictly symmetrical) and the resolution step (symmetrical due to the RuvC action). The high proportion of symmetrical products, combined with the 100-fold lower efficiency of recombination for 22% as compared to 4% diverged sequences, may suggest the existence of two recombination mechanisms inherent to the RecABCD pathway: one being prominent at low sequence divergence (non-reciprocal), and the other at high levels of divergence (crossover). In line with this, we found that RecABCD-promoted recombination was independent of RuvABC at low divergence, but depended on RuvABC for the 22% diverged DNA. The prevalence of crossovers at high divergence might result from a combination of two favouring conditions: i) the requirement of a single event, rather than two for the non-reciprocal recombination, ii) the relative higher stability of highly mismatched heteroduplexes within Holliday junctions, as compared to the non-reciprocal recombination intermediates.

This reasoning, in turn, underlines again the different activity of Red proteins, which produce mainly (81% of cases) non-symmetrical recombinants between 22% diverged sequences. Still, the probability that the observed 17% symmetrical products were generated by chance during two successive non-reciprocal exchanges occurring in the same of the 137 possible intervals is very low. We propose that a fraction (∼2×17% = 34%) of all Red promoted events in our experimental set up are indeed cross-overs. Biochemical studies of the RecT protein, which belongs to the same family as Redβ, have suggested that it might be able to generate three-strand intermediates [53]. *In vivo*, both reciprocal and non-reciprocal events are promoted by Red enzymes, and the balance is given by the length of homology available at the broken extremity: the longer the homology, the more non-reciprocal events are made [52].

Furthermore, the detailed analysis of joints produced via the Rec and Red pathways between 22% diverged DNA suggests again mechanistic differences which are compatible with the biochemical properties of the two systems: two hot spots are observed for the Rec products. RecA-promoted homologous recombination is expected to act more or less equally on all DNA sequences, but the absence of any single identical interval large enough to accommodate a MEPS (minimal efficient pairing sequence, [22]) may force the appearance of preferred regions where the three strand intermediate had a better stability. Indeed, a detailed study of the effect of mismatches on RecA-mediated joint molecule formation has shown that the position of mismatches relative to the identical regions can have different effects, depending on the stability of the heteroduplex progressively formed as exchange proceeds [54]. In contrast, no such hot spot is seen with Red products, which may well correspond to a ‘sandwich-like’ mode of action of single-strand annealing enzymes, rather than the progressive invasion process mediated by RecA.

### λ Recombination as a Tool for Biotechnology Applications

We demonstrate that, starting from pairs of similar genes, irrespective of their origin, phage genetic promiscuity can be exploited to generate large new gene families creating potentially interesting new biochemical entities. Even at 22% divergence, the Red recombination pathway can routinely create 10^5^ to 10^6^ recombinant genes (and viruses) per single Petri dish, 40% of which represent different new genes. The yield of recombinant genes is orders of magnitude higher than when the same genes were carried in *E. coli* plasmids [55]. It is also possible to lead this system through unlimited iterative cycles of inversion recombination, which should yield even more diverse gene products. As such, λ is therefore a convenient genetic vector for evolutionary biotechnology.

### Homeologous Recombination and the Origin of Temperate Virus Genomic Mosaicism

Can we relate our experimental results on homeologous recombination in λ to the evolutionary history of lambdoid virus genomes? Our bioinformatic analysis showed that among all detected blocks of highly similar sequences (hits), about one half showed no flanking “shoulder” of moderate divergence, about 40% showed only one shoulder and the remaining hits were clearly framed by two shoulders. Even a single shoulder is compatible with an involvement of homeologous recombination. For example, a sequence block can be acquired by an homeologous recombination event (shoulder) at one junction, accompanied by an homologous event between identical sequences [9] or an illegitimate event at the second junction (no shoulder, [45]).

When shoulders were detected, their identity was in the range of 64% to 68%, and the hit sequences were on the average 94% identical. The 6% divergence of the hit sequence suggests that, at the time of recombination, the shoulders identity was about 70 to 74% (Figure 5). This is close to the 78% identity that was tested in our assay and found as substrate for homeologous recombination. Because never more than 50% of the detected hits were flanked by at least one recognizable shoulder, illegitimate recombination and homeologous recombination appear to contribute to phage genomic mosaicism to a similar extent.

If virus mosaicism is really related to the presence of Redβ-like recombination enzymes, it should be possible to verify that all virus genomes exhibiting mosaicism encode such a function. Among the ten lambdoids from enterobacteria that were analysed here, only two encoded a Redβ ortholog. However at least one other family of virus recombinases, of which Erf is the best studied member, has been described [47]. It may act similarly to Redβ, as it forms similar ring structures [56], and cross-complementation has been observed [57]. Four among the ten lambdoids from enterobacteria encode an Erf ortholog, and eight among the fifteen lambdoids from lactic acid bacteria as well. Whether this type of recombinase promotes efficient homeologous recombination remains to be tested. None of the *S. aureus* virus analysed encode either a Redβ or Erf ortholog. It may be that one or more virus recombinase families remain unknown at present. Interestingly, viruses belonging to the family of T4, composed exclusively of virulent members, appear not to have a mosaic structure, but to consist rather, like bacteria, in a common backbone genome, interrupted by a few large variable regions [58]. These viruses do not encode proteins of the Redβ nor Erf family, but a UvsX protein which has ATPase activity like RecA. Also, among dairy viruses, a different genomic structure for virulent and temperate viruses has been reported [59]. This scattered evidence is therefore compatible with the possibility that the mosaicism of lambdoid genomes is connected with the particular type of homologous recombination enzymes they encode, which may be fit to provide, in a short time, large gene repertoires and therefore bring about an extraordinary evolvability.

## Materials and Methods

### Strains

All *Escherichia coli* and λ strains used in this study are described in Table 4.

**Table 4:** Strains and plasmids used in this study (All *E. coli* strains, unless specified otherwise, are derivatives of C600).

Strain	Genotype	Source/construction
*E. coli*		
C600	*thr1 leuB6 thi1 lacY1 supE44 rfbD1 fhuA21*	E. *coli* Gen. Stock Cr
NK5196	P2 prophage	[23]
GSY 579	AB1157 lysogen for λ*c*I*857ts*	S. Sommer
GSY5902	AB1157 *ΔrecA306 srl::*Tn*10 (miniF recA)*	S. Sommer
MAC 49	AB1157 *mutS215*::Tn*10*	G. Walker
NK7085	AB1157 *mutS104::*Tn*5*	N. Kleckner
NEC58	AB1157 *recQ61::*Tn*3*	Laboratory collection
FR189	AB1157 *uvrD::*Tn*5*	Laboratory collection
MAC833	AB1157 *uvrD:*:PhleoR	[26]
JJC754	AB1157 *ruvABC:CmR*	B. Michel
JTM93	*ΔrecA306 srl:*:Tn*10*	P1 GSY5902*C600
MAC999	*mutS104::*Tn*5*	P1 NK7085*C600
JTM304	*recQ61::*Tn*3*	P1 NEC58*C600
JTM322	*uvrD::*Tn*5*	P1 FR189*C600
MAC 1256	*ruvABC*:CmR	P1 JJC754 * C600
JTM94	P2 prophage *mutS215:*:Tn*10*	P1 MAC49*NK5196
JTM159	*mutS::*Tn*5 recA306srl::*Tn*10*	P1 GSY5902*MAC999
JTM334	P2 prophage *recQ61::*Tn*3*	P1 NEC58*NK5196
JTM335	*recQ61::*Tn*3 recA306srl::*Tn*10*	P1 GSY5902*JTM304
JTM336	P2 prophage *uvrD:*:PhleoR	P1 MAC833*NK5196
JTM337	*uvrD::*Tn*5 recA306srl::*Tn*10*	P1 GSY5902*JTM322
MAC1259	P2 prophage *ruvABC:*:CmR	P1 JJC754*NK5196
MAC 1266	*ruvABC:*:CmR *recA306srl::*Tn*10*	P1 GSY5902*MAC1256
JTM146	AB1157 (λ*c*I*857ts*) *pKD46*	pKD46 into GSY579
λ *strains*		
1390	*b221 red3 ea10:*IS10 *rexA*:Tn*10* del 267 inv (pL-N) *c*I*857ts* χ+C	[24]
Nec1	*ea10:oxa7 rexA:oxa7*-CmR inv (pL-N) χ+ *c*I*857ts Δorf28-ral*	This work
Nec2	*ea10:oxa7 rexA:oxa11*-CmR inv (pL-N) χ+ *c*I*857ts Δorf28-ral*	This work
Nec3	*ea10:oxa7 rexA:oxa5*-CmR inv (pL-N) χ+ *c*I*857ts Δorf28-ral*	This work
Nec4	*ea10:oxa7 rexA:oxa7*-CmR χ+ *c*I*857ts Δorf28-ral*	This work
Nec5	*ea10:oxa7-11 rexA:oxa11-7*-CmR χ+ *c*I*857ts Δorf28-ral*	This work
Nec6	*ea10:oxa7-5 rexA:oxa5-7*-CmR χ+ *c*I*857ts Δorf28-ral*	This work
Nec8	*ea10:oxa7 rexA:oxa1*-PhleoR χ+ *c*I*857ts Δorf28-ral*	This work
*Plasmid*		
pKD46	λ *red gam* expressing plasmid	[61]

### General Phage Manipulations

Lysogenization was performed as described by Cromie and colleagues [60]. Primary phage stocks, which all contained the thermosensitive *c*I*857ts* mutation, were obtained by shifting cultures of lysogenic bacteria at a OD_600_ 0.4 for 10 minutes to 45°C, followed by further incubation (up to 4 hrs) at 37°C. These primary stocks usually contained 10^10^ plaque forming units per ml.

### λ Phage Construction

The λ 366 described by N. Kleckner [23] contained a copy of IS10 inserted into the *ea10* gene (our unpublished observation), and a copy of *Tn*10 inserted into the *rexA* gene. A derivative obtained by G. Smith, λ 1390, in which the pL promoter is inverted, was used as the starting material for our constructions [24]. Our goal was to replace the IS10 and *Tn*10 copies by a set of related *oxa* genes which diverge by 4% (between *oxa7* and *oxa11*), 22% (between *oxa7* and *oxa5*), or 52% (between *oxa7* and *oxa1*, [55]). A fragment of the λ1390 genome was cloned onto plasmid pACYC184, and successive cloning steps allowed to substitute part of the IS10 with *oxa7*, and the totality of *Tn*10 with three elements: i) either *oxa7*, *oxa11,* or *oxa5*, inverted relative to the copy of *oxa7* inserted into *ea10*, ii) the chloramphenicol-resistance (cm^R^) gene of pACYC184, and iii) a Chi site [24]. Integration of these cassettes into λ*c*I*857ts* was done using the protocole of Datsenko and Wanner [61], with strain JTM146 as a recipient. This permitted to obtain λNec1, 2, 3, in which the pL promoter is inverted. To get the inversed orientation of pL, recombinants obtained starting from λ Nec1, 2, and 3 constructions were selected, and a clone in which the recombinant product was symmetrical was kept. The construction to test 52% diverged sequences in the Red pathway (λNec8) was done by replacing the rightward oxa7-CmR cassette of λNec4 by an oxa1-phleoR cassette. Construction details are available in Text S1 and Figure S3.

The construction to test 52% diverged sequences in the Red pathway (λNec8) was done by replacing the rightward *oxa7*-CmR cassette of λNec 4 by an *oxa1*-PhleoR cassette. To do this, a plasmid containing the *c*I to N region of λ, in the native orientation of the pL promoter, interrupted by the *oxa*5-CmR cassette (pMAP189) was used to substitute a different cassette, made of the *oxa*1 gene flanking a PhleoR gene, giving plasmid pMAP195. The 3.2 kb *Ava*II-*Sap*I fragment of pMAP195 was then gel purified and used to transform a C600 derivative lysogenic for λ Nec4 and containing pKD46, and selecting phleomycin resistant transformants (1 µg/ml), in which the rightward *oxa*7-CmR cassette had been replaced by the *oxa*1-phleoR cassette.

### Recombination Measurements

#### Single Step Experiments

Phages were adsorbed on the selective host at an m.o.i. of 0.1 for 30 minutes at 37°C. Infected cells were diluted 100-fold in pre-warmed TB (10 g/L Bacto-Tryptone, 0.5% w/v NaCl) supplemented with 0.1% maltose and 1 µg/ml thiamin, and grown at 37°C for 3.5 h when the first peak of phage production occurred. The supernatant was collected, filter-sterilized, and phage stocks were titrated on C600 *recA* and C600 P2. Recombinant frequency was calculated by determining the ratio of phages growing on the lawn selective for recombinants, over total phage count estimated by the sum of titers obtained on P2 and *recA* lawns.

#### Confluent Phage Growth

Recombination frequencies were estimated on phage stocks grown on plates to confluence, starting with an m.o.i. of 0.1. As Red^−^ Gam^−^ phages have lower burst sizes compared to Red^+^Gam^+^ phages in a wild type host, growth was performed under restrictive conditions, such that recombined phages could not propagate. This prevented possible enrichment, allowed us to measure the yield of recombinants produced during the last burst before phage harvest, and to deduce a recombinant frequency per generation. 100 µl of an over night (ON) culture of the respective host bacteria (i.e. C600 *recA* or its *mutS, uvrD, ruvABC* or *recQ* derivatives for Red-mediated recombination of λ; and C600 (P2) or its *mutS, uvrD, ruvABC* or *recQ* derivatives for RecABCD-mediated recombination of λ) were mixed with 100 µl of primary stock phages in 5 ml of top agar (10 g/l Bacto-Tryptone, 4.5 g/l Bact-Agar, 0.25% w/v NaCl, 10 mM MgSO4), and the mixture poured on LB plates. The plates were incubated ON at 37°C. Top agar was harvested and mixed with 3 ml Suspension Medium (SM: 50 mM Tris·Cl, pH 7.5 at RT; 0.1 M NaCl; 8 mM MgSO4; 0.01% gelatin). The mixture was centrifuged and the supernatant titrated as for single step experiments.

Background level of *gam* ^−^ phages due to mutation was measured by plating a λ*c*I*857ts* strain on a P2 lysogen, and found to be 5×10^−7^. Therefore, contribution of mutagenesis to the scoring of Red-dependent, *gam-* recombinants was considered negligible.

**Figure 6:**
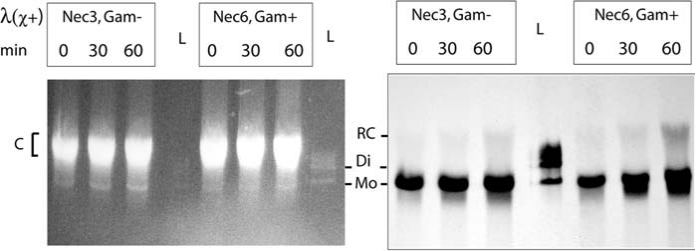
Both λ Nec3 and λ Nec6 produce rolling circle intermediates. DNA extracted during a kinetics of λ infection was loaded on a gel (30 µl), besides a λ ladder size marker (shown as L, 1 ng in the middle lane, 10 ng on the right side lane). UV photograph of the gel is shown in the left panel, and Southern blot in the right panel (the last lane was omitted for blotting). Mo, Di and RC: monomer, dimer and rolling-circle forms of λ respectively. C: *E. coli* C600 chromosomal DNA.

λ replicates by two distinct modes, theta type and rolling-circle type, which may be different substrates for recombination. However, all derivatives analysed in this work contain a Chi site, so they should produce rolling circle intermediates, even in the absence of Gam, as is the case when pL is inverted. We verified by Southern analysis that both types of constructs, with pL inverted or not, produced rolling circle intermediates in our growth conditions. To do so, phages were adsorbed to 1 ml of C600 cells grown to an OD of 0.5 (in TB medium supplemented with 0.1% maltose and thiamin 1 µg/ml), at an MOI of 1, at 37°C without agitation. Samples were withdrawn 0, 30 and 60 minutes after adsorption, cells were pelleted and resuspended in 100 µl of SET buffer (20% sucrose, 50 mM Tris pH 7.5, 50 mM EDTA, 0.5 mg/ml lysozyme), and incubated 10 min at 37°C. Lysis was then completed by adding 100 µl of SET supplemented with 5% SDS and bromophenol blue. Crude extracts were vortexed 1 minute, and loaded (30 µl) on a 15 cm-long, 0.5% TBE agarose gel supplemented with 40 µg/ml ethidium bromide. To achieve best separation, electrophoresis proceeded in TBE buffer with 40 µg/ml ethidium bromide for 3 h at 150 volts. This high migration voltage heated considerably both buffer and gel, and this appeared necessary to achieve best resolution, as the same gels run in the cold room did not allow to separate λ from the bulk of chromosomal DNA as nicely. Under such conditions, the dimer and trimer of λ, prepared by partial ligation, migrated faster than the rolling-circle intermediates, which co-migrated with the upper limit of the bulk of chromosomal DNA. Transfer, and hybridisation, followed classical protocols (the whole λ genome was taken as a probe). Results on Figure 6, right panel, show that both λNec3 (Gam^−^) and λNec6 (Gam^+^) produce rolling circle intermediates as a function of time, with the Gam- phage producing less than the Gam+ phage, as expected. Monomeric molecules migrate ahead of rolling-circle products, and dimer molecules of λ are absent. A similar result was obtained when phages were adsorbed to the strains used for recombination scoring (P2 lysogen for Nec3, and *recA* mutant for Nec6).

### Sequencing of Recombinants

Single plaques of recombinants were purified by streaking, purified plaques were toothpicked and resuspended in SM. These crude phage particles were directly used for PCR amplification with oligonucleotides flanking the *oxa* gene to be sequenced. When the same pairs of oligonucleotides were used on the starting, non-inverted phages, no PCR product was obtained, ensuring that the recombinants analysed were not generated during the PCR itself.

### Computational Analysis

#### Shoulder Detection Strategy

A flowchart is given as Figure S2. For each pair of bacteriophage genomes, a blast allowing gaps was run, and all hits longer than 200 bp, having an E value lower than 10^−8^ and exhibiting more than 90% nucleotide identity, were kept for further analysis. For each hit, the pairs of left- and right- flanking DNA fragments were aligned using the Needleman-Wunsch algorithm. The size of the analyzed flanking region was 2 kb, with an additional 200 bp-long “anchor” inside the hit. In cases where two hits were closer than 2 kb apart on one or both genomes, the flanking fragment size was set to the size of the smaller inter-hit intervals, and alignment was calculated only if fragments were longer than 100 bp. The alignment result was converted into a vector storing the percentage of identity (idperc) in each 100nt-long interval. The hit idperc value (called *h*) was the integer value of the blast output. The background level of idperc (called *b*) for a given genome pair was estimated by pooling values obtained in all vectors of this pair, and extracting the one third median. A shoulder was then defined as any interval of the vector (at least 100 nt long), directly flanking the hit, and in which all idperc values *s*, were such that *b*+10<*s*<*h*−10. Some flexibility was added to this rule, so as to permit any of the *s* values to be less than *b*+10, provided that its two neighbors were more than *b*+10.

#### Calculation of Shoulder Number Expected at Random

Vectors calculated as described above were used to detect regions similar to the shoulders in terms of idperc, but not placed at the flanking side of the hit. These heterogeneities will be called tentatively “bumps”. Cumulated bump length found at the vicinity of all hits, divided by the total length scanned (over all vectors of all hits), allowed to estimate the bump density. This density was then multiplied by the cumulated shoulder lengths, to give an estimation of the number of shoulders that correspond in fact to the background “noise” of heterogeneity of all alignments.

#### Construction of Phages Families and Informatic Tools

Ten genomes of lambdoid phages from enterobacteria were used: λ, HK022, HK97, P22, 933W, HK620, N15, P27, D3, APSE. The set of 15 lactic acid bacteria infecting phages was selected according to Proux et al. (Proux et al., 2002): A2, Tuc2009, BK5-T, TP901-1, r1t, bIL67, c2, sk1, bIL170, bIL309, bIL285, bIL286, 7201, sfi19, sfi21. The set of 20 *S. aureus* bacteriophages was selected according to Kwan et al. (Kwan et al., 2005): 187, 69, 53, 85, 2638A, 77, 42e, 3A, 47, 37, EW, 96, ROSA, 71, 55, 29, 52A, 88, 92, X2. Scripts were written in Python, using the Biopython parser for genbank files. BLAST 2.2.10 was downloaded from the NCBI.

## Supporting Information

Figure S1 Nucleotide sequence alignment of oxa genes used as recombination substrates. (A) oxa7 to oxa11 (4% divergence). (B) oxa7 to oxa5 (22% divergence). Alignments were performed with the Needleman-Wunsch algorithm.
(0.03 MB DOC)

Figure S2 Flowchart of the shoulder detection strategy.
(0.02 MB DOC)

Figure S3 Sketch of the different steps of the Lamda Nec contructions.
(0.21 MB DOC)

Table S1 Joints positions in recombinants obtained with 22% diverged DNA.
(0.02 MB XLS)

Table S2 Position of the mosaics detected among the enterobacteria bacteriophages.
(0.03 MB XLS)

Table S3 Position of the mosaics detected among lactic acid bacteria bacteriophages.
(0.04 MB DOC)

Table S4 Positions of the mosaics detected among Staphylococcus aureus bacteriophages.
(0.14 MB DOC)

Text S1 Construction of the Lambda Nec series.
(0.03 MB DOC)
