# Supplementary material for: The λ Red Proteins Promote Efficient Recombination between Diverged Sequences: Implications for Bacteriophage Genome Mosaicism
Source: PLoS Genet. 2008 May 2;4(5):e1000065. doi: 10.1371/journal.pgen.1000065 (PMC2327257; doi:10.1371/journal.pgen.1000065)
Supplement: Figure S1 — Nucleotide sequence alignment of oxa genes used as recombination substrates. (A) oxa7 to oxa11 (4% divergence). (B) oxa7 to oxa5 (22% divergence). Alignments were performed with the Needleman-Wunsch algorithm. (0.03 MB DOC) [file pgen.1000065.s001.doc]

**Figure S1**

**A)**

oxa7 1 ATGAAAACATTTGCCGCATATGTAATTACTGCGTGTCTTTCAAGTACGGCATTAGCTAGTTCAATTACAGAAAATACGTTTTGGAACAAAGAGTTCTCTG

||||||||||||||||||||||||||||..|||||||||||.|||||||||||||||.|||||||||||||||||||||.||||||||||||||||||||

oxa11 1 ATGAAAACATTTGCCGCATATGTAATTATCGCGTGTCTTTCGAGTACGGCATTAGCTGGTTCAATTACAGAAAATACGTCTTGGAACAAAGAGTTCTCTG

oxa7 101 CCGAAGCCGTCAATGGTGTTTTCGTGCTTTGTAAAAGTAGCAGTAAATCCTGCGCTACCAATAACTTAGCTCGTGCATCAAAGGAATATCTTCCAGCATC

|||||||||||||||||||.||||||||||||||||||||||||||||||||||||||||||.|||||||||||||||||||||||||||||||||||||

oxa11 101 CCGAAGCCGTCAATGGTGTCTTCGTGCTTTGTAAAAGTAGCAGTAAATCCTGCGCTACCAATGACTTAGCTCGTGCATCAAAGGAATATCTTCCAGCATC

oxa7 201 AACATTTAAGATCCCCAACGCAATTATCGGCCTAGAAACTGGTGTCATAAAGAATGAGCATCAGATTTTCAAATGGGACGGAAAGCCAAGAGCCATGAAA

||||||||||||||||||||||||||||||||||||||||||||||||||||||||||||||||.||||||||||||||||||||||||||||||||||.

oxa11 201 AACATTTAAGATCCCCAACGCAATTATCGGCCTAGAAACTGGTGTCATAAAGAATGAGCATCAGGTTTTCAAATGGGACGGAAAGCCAAGAGCCATGAAG

oxa7 301 CAATGGGAAAGAGACTTGAGCTTAAGAGGGGCAATACAAGTTTCAGCGGTTCCCGTATTTCAACAAATCGCCAGAGAAGTTGGCGAAGTAAGAATGCAGA

|||||||||||||||||||.|||||||||||||||||||||||||||.||||||||||||||||||||||||||||||||||||||||||||||||||||

oxa11 301 CAATGGGAAAGAGACTTGACCTTAAGAGGGGCAATACAAGTTTCAGCTGTTCCCGTATTTCAACAAATCGCCAGAGAAGTTGGCGAAGTAAGAATGCAGA

oxa7 401 AATATCTTAAAAAATTTTCATATGGTAACCAGAATATCAGTGGTGGCATTGACAAATTCTGGTTGGAGGGTCAGCTTAGAATTTCCGCAGTTAATCAAGT

||||.||||||||||||||.|||||.|.|||||||||||||||||||||||||||||||||||||||.|..|||||||||||||||||||||||||||||

oxa11 401 AATACCTTAAAAAATTTTCCTATGGCAGCCAGAATATCAGTGGTGGCATTGACAAATTCTGGTTGGAAGACCAGCTTAGAATTTCCGCAGTTAATCAAGT

oxa7 501 GGAGTTTCTAGAGTCTCTATTTTTAAATAAATTGTCAGCATCAAAAGAAAATCAGCTAATAGTAAAAGAGGCTTTGGTAACGGAGGCTGCGCCTGAATAT

||||||||||||||||||||.|||||||||||||||||||||.||||||||.|||||||||||||||||||||||||||||||||||.||.|||||||||

oxa11 501 GGAGTTTCTAGAGTCTCTATATTTAAATAAATTGTCAGCATCTAAAGAAAACCAGCTAATAGTAAAAGAGGCTTTGGTAACGGAGGCGGCACCTGAATAT

oxa7 601 CTTGTGCATTCAAAAACTGGTTTTTCTGGTGTGGGAACTGAGTCAAATCCTGGTGTCGCATGGTGGGTTGGTTGGGTTGAGAAGGGAGCAGAGGTTTACT

||.||||||||||||||||||||||||||||||||||||||||||||||||||||||||||||||||||||.|||||||||||||...||||||||||||

oxa11 601 CTAGTGCATTCAAAAACTGGTTTTTCTGGTGTGGGAACTGAGTCAAATCCTGGTGTCGCATGGTGGGTTGGGTGGGTTGAGAAGGAGACAGAGGTTTACT

oxa7 701 TTTTCGCCTTTAACATGGATATAGACAACGAAAATAAGTTGCCGCTAAGAAAATCCATTCCCACCAAAATCATGGCAAGTGAGGGCATCATTGGTGGCTAA

|||||||||||||||||||||||||||||||||.|||||||||||||||||||||||||||||||||||||||||.|||||||||||||||||||||||||

oxa11 701 TTTTCGCCTTTAACATGGATATAGACAACGAAAGTAAGTTGCCGCTAAGAAAATCCATTCCCACCAAAATCATGGAAAGTGAGGGCATCATTGGTGGCTAA

**B)**

oxa7 1 ATGAAAACATTTGCCGCATATGTAATTACTGCGTGTCTTTCAAGTACGGCATTAGCTAGTTCAATTACAGAAAATACGTTTTGGAACAAAGAGTTCTCTG

||||||||..|.|||||||||.||.||...|..|.|..|.||||.||.||..|..|....||.|||.|.||||||..|...|||||.|||||.||.|||.

oxa5 1 ATGAAAACCATAGCCGCATATTTAGTTCTAGTTTTTTATGCAAGCACCGCGCTCTCAGAGTCTATTTCTGAAAATTTGGCGTGGAATAAAGAATTTTCTA

oxa7 101 CCGAAGCCGTCAATGGTGTTTTCGTGCTTTGTAAAAGTAGCAGTAAATCCTGCGCTACCAATAACTTAGCTCGTGCATCAAAGGAATATCTTCCAGCATC

..|||.||||..||||.|||||.||.||||||||||||||.||.||.|||||..||||.|||||....||.||||||||.|..|..|||.||||||||||

oxa5 101 GTGAATCCGTACATGGCGTTTTTGTACTTTGTAAAAGTAGTAGCAATTCCTGTACTACAAATAATGCGGCACGTGCATCTACAGCCTATATTCCAGCATC

oxa7 201 AACATTTAAGATCCCCAACGCAATTATCGGCCTAGAAACTGGTGTCATAAAGAATGAGCATCAGATTTTCAAATGGGACGGAAAGCCAAGAGCCATGAAA

||||||.||.||.||.||.||..|.||.||.||.|||||.||.|.||||||..||||.|..|||.||||||||||||||||.|||||.|||||||||||.

oxa5 201 AACATTCAAAATTCCTAATGCTCTAATAGGTCTTGAAACCGGCGCCATAAAAGATGAACGGCAGGTTTTCAAATGGGACGGCAAGCCCAGAGCCATGAAG

oxa7 301 CAATGGGAAAGAGACTTGAGCTTAAGAGGGGCAATACAAGTTTCAGCGGTTCCCGTATTTCAACAAATCGCCAGAGAAGTTGGCGAAGTAAGAATGCAGA

||||||||||.||||||.|...||||.||.||.|||||.|||||.||.|||||.||||||||||||||.||||||||||||||||||.||||||||||.|

oxa5 301 CAATGGGAAAAAGACTTAAAGCTAAGGGGCGCTATACAGGTTTCTGCTGTTCCGGTATTTCAACAAATTGCCAGAGAAGTTGGCGAAATAAGAATGCAAA

oxa7 401 AATATCTTAAAAAATTTTCATATGGTAACCAGAATATCAGTGGTGGCATTGACAAATTCTGGTTGGAGGGTCAGCTTAGAATTTCCGCAGTTAATCAAGT

||||.|||||....||||||||.||.|||...|||||..|.||.||||||||||||||||||.|.||.||||||||||||||.||.|||.|.||||||||

oxa5 401 AATACCTTAACCTGTTTTCATACGGCAACGCCAATATAGGGGGAGGCATTGACAAATTCTGGCTAGAAGGTCAGCTTAGAATCTCAGCATTCAATCAAGT

oxa7 501 GGAGTTTCTAGAGTCTCTATTTTTAAATAAATTGTCAGCATCAAAAGAAAATCAGCTAATAGTAAAAGAGGCTTTGGTAACGGAGGCTGCGCCTGAATAT

..|.|||.|||||||.||.|...|.|||||.|||.||||||||||||.|||.||.|||||||||||||||||..|.||.||.||.||..|.||.||||||

oxa5 501 TAAATTTTTAGAGTCGCTCTACCTGAATAATTTGCCAGCATCAAAAGCAAACCAACTAATAGTAAAAGAGGCAATAGTTACAGAAGCAACTCCAGAATAT

oxa7 601 CTTGTGCATTCAAAAACTGGTTTTTCTGGTGTGGGAACTGAGTCAAATCCTGGTGTCGCATGGTGGGTTGGTTGGGTTGAGAAGGGAGCAGAGGTTTACT

.|.||.||||||||||||||.|.|||.|||||.||.||.||.||||.||||||||||||.|||||||||||||||||.|||||.|||.|.||||||||||

oxa5 601 ATAGTTCATTCAAAAACTGGGTATTCCGGTGTTGGCACAGAATCAAGTCCTGGTGTCGCTTGGTGGGTTGGTTGGGTAGAGAAAGGAACTGAGGTTTACT

oxa7 701 TTTTCGCCTTTAACATGGATATAGACAACGAAAATAAGTTGCCGCTAAGATGGTGTCGCTTGGTGGGTTGGTTGGGTAGAGAAAGGAACTGAGGTTTACT

||||.||.|||||||||||.||||||||.||.|.|||.||||||..|||||||||||||.|.||.|||||||||||||||||.|| ||||||||||||

oxa5 701 TTTTTGCTTTTAACATGGACATAGACAATGAGAGTAAATTGCCGTCAAGAAAATCCATTTCAACGAAAATCATGGCAAGTGAAGGCATCATCATTGGTGG

oxa7 801 CTAA

||||

oxa5 801 CTAA

**Fig.** **S1 legend.** Nucleotide sequence alignment of *oxa* genes used as recombination substrates. (A) *oxa7* to *oxa*11 (4% divergence). (B) *oxa7* to *oxa*5 (22% divergence). Alignments were performed with the Needleman-Wunsch algorithm.
