# Supplementary material for: The λ Red Proteins Promote Efficient Recombination between Diverged Sequences: Implications for Bacteriophage Genome Mosaicism
Source: PLoS Genet. 2008 May 2;4(5):e1000065. doi: 10.1371/journal.pgen.1000065 (PMC2327257; doi:10.1371/journal.pgen.1000065)
Supplement: Figure S2 — Flowchart of the shoulder detection strategy. (0.02 MB DOC) [file pgen.1000065.s002.doc]

Flowchart of the shoulder detection strategy

For all pairs of genomes in a phage family,

Run a blast

Select hits with a length>200 bp and %identity >90%

For each hit, extract the pair 2kb of neighbor sequences on both sides, and generate a Needleman and Wunsch (NW) alignment

On each NW alignement,

Use a 100 bp sliding window to estimate % identity in each 100 bp interval

Retain as ‘shoulders’ the part of the alignment flanking the hit where the

%identity is above the background %identity by at least 10 points.
