# Supplementary material for: The λ Red Proteins Promote Efficient Recombination between Diverged Sequences: Implications for Bacteriophage Genome Mosaicism
Source: PLoS Genet. 2008 May 2;4(5):e1000065. doi: 10.1371/journal.pgen.1000065 (PMC2327257; doi:10.1371/journal.pgen.1000065)
Supplement: Text S1 — Construction of the Lambda Nec series. (0.03 MB DOC) [file pgen.1000065.s008.doc]

**Supplementary Text S1**

**Construction of the λ Nec series**

A sketch of the principal steps of plasmid constructions is shown in Supplementary Figure S3. The 3.8 kb *Fsp*I fragment of λ1390, encompassing IS*10* flanked by the pL-*N* inversion on one side, and the *gam-bet* genes on the other, was cloned into the *Xmn*I site of pACYC184. Then *oxa7*, the oxacillin hydrolysing beta-lactamase gene carried by plasmid pMG202 of *E. coli*, was amplified by PCR from plasmid pMIX93, and substituted to IS10 and the *rexB* gene, between the *Pvu*I and *Nde*I sites. This resulted in the ‘left arm’ plasmid, pAC11oxa7 (Fig. S3, left part). To construct the right side arm vector, the 1 kb *c*I*-rexA* region of λ (nt 36846-37844 of λ), was amplified by PCR on λ using an oligonucleotide providing the 8-pb Chi site properly oriented on the *rexA* side, and introduced between the *Ava*I and *Bsu*36I sites of pACYC184, to give pMAP174. In this vector, the various *oxa* genes were introduced: *oxa7*, cloned from pMIX93, was inserted between sites *Xmn*I and *Sca*II of pMAP174, *oxa11*, the gene from plasmid pMLH502 of *Pseudomonas aeruginosa*, was PCR-amplified from pMIX95 [55] and inserted between sites *Xmn*I and *Sca*II of pMAP174 and *oxa*5, the gene from pMG54 of *P. aeruginosa*, was PCR-amplified from pMIX96 [55], and inserted between sites *Xmn*I and *Bst*1107I of pMAP174. An additional 40 bp deletion (*Bst*1107I-*Bst*XI) was effected on the *oxa7* and *oxa*11 derivatives, to prevent formation of a small inverted repeat flanking the left side *oxa7* in the final construct. These three ‘right arm plasmids’ (Figure S3, right part), pMAP184, 185 and 186, were then assembled with the ‘left arm’ pAC11oxa7 plasmid as follows. Plasmids pMAP184, 185 and 186 were opened with *Sac*II, and the ‘left arm region’ between sites *Bst*1107I and *Psi*I was inserted into it, giving rise to plasmids pACλ7-7, pMAP187 and pMAP188 respectively. The 6 kb long *Sal*I-*Eco*O109I fragment of these plasmids was gel-purified and used directly for transformation by electroporation in strain JTM 146, containing pKD46 and lysogenic for λ, using the protocol of Datsenko and Wanner [61]. The fragments contained a 1.2 kb homology on its left side, and a 1 kb homology on its right side, with the resident λ prophage.
