# Supplementary material for: The λ Red Proteins Promote Efficient Recombination between Diverged Sequences: Implications for Bacteriophage Genome Mosaicism
Source: PLoS Genet. 2008 May 2;4(5):e1000065. doi: 10.1371/journal.pgen.1000065 (PMC2327257; doi:10.1371/journal.pgen.1000065)
Supplement: Supplementary file 8 [file pgen.1000065.s003.doc]

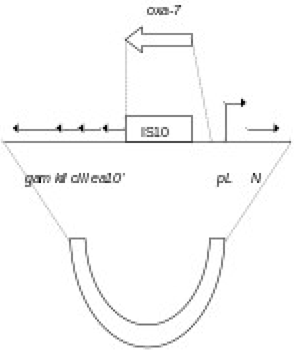

'Left-arm' plasmid

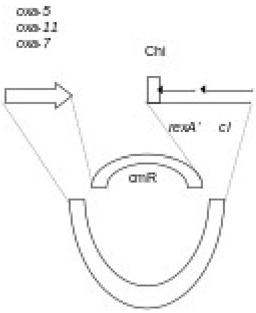

'Right-arm' plasmid
